# Tai Chi Training and Pre-Competition Anxiety in High-Level Competitive Athletes: A Chain Mediation Model of Flow and Mental Toughness

**DOI:** 10.3390/bs16020163

**Published:** 2026-01-23

**Authors:** Runze Guo, Jing Liu

**Affiliations:** College of Wushu, Shanghai University of Sport, Shanghai 200438, China; 2011212007@sus.edu.cn

**Keywords:** Tai Chi training, pre-competition anxiety, high-level competitive athletes, flow experience, mental toughness

## Abstract

With the increasing competition in elite sports, pre-competition anxiety has become increasingly prevalent among high-level competitive athletes, and high levels of such anxiety may impair sports performance and threaten athletes’ psychological health. Traditional psychological interventions (e.g., cognitive-behavioral therapy) are often poorly accepted and costly; however, pre-competition anxiety in these athletes may be alleviated through multiple pathways of traditional mind–body exercises like Tai Chi. Yet, the psychological mechanism by which mind–body exercises such as Tai Chi training influence pre-competition anxiety remains insufficiently explored, particularly the chain-mediating effect of the “flow experience → mental toughness” pathway. This study thus aimed to investigate the impact of Tai Chi training on pre-competition anxiety in high-level competitive athletes and verify the chain-mediating role of the “flow experience → mental toughness” pathway, thereby providing a theoretical basis and practical reference for sports psychology interventions. Using a randomized controlled experimental design, 86 high-level competitive athletes were randomly divided into an experimental group (n = 43) and a control group (n = 43). The experimental group received standardized Tai Chi training for 8 weeks, while the control group maintained their regular training regimen. Data were collected at baseline, week 4, and week 8 of the intervention using the Competition State Anxiety Inventory-2 (CSAI-2), Flow State Scale-2 (FSS-2), and Sport Mental Toughness Questionnaire (SMTQ), and chain-mediating effects were tested via hierarchical regression analysis and the bootstrap method with 5000 resamples. The results indicated that Tai Chi training could reduce pre-competition anxiety levels (β = −0.30, *p* < 0.5), and both flow experience (β = 0.38, *p* < 0.5) and mental toughness (β = 0.21, *p* < 0.5) exerted significant mediating effects. The chain mediation model further revealed that Tai Chi training alleviated pre-competition anxiety by enhancing flow experience and improving mental toughness sequentially (β = 0.01, 95% CI [0.00, 0.03]), accounting for 78.9% of the total mediated effect. In conclusion, Tai Chi training is associated with reduced pre-competition anxiety in high-level competitive athletes, and this relationship is statistically mediated by the sequential pathway of flow experience and mental toughness. These findings offer a new theoretical basis and practical direction for mind–body interventions in sports psychology. It should be noted that future research could further optimize and refine the intervention protocol, and explore the underlying mechanism of mind–body interventions at the neurobiological level.

## 1. Introduction

Competitive sports demand high levels of psychological stress to produce high-level athletes, and pre-competition anxiety. Multidimensional Anxiety Theory (MAT) defines it as a multidimensional psychological condition (comprising cognitive anxiety (worry, negative thoughts), somatic anxiety (physiological arousal), and low self-confidence of state). All these dimensions have an impact on the performance and the mental health of the athletes (Martens et al., 1990). Although the present research delves into the international impact of Tai Chi on pre-competition anxiety, we are still sensitive to this multidimensional model, and we will report on the results of the individual subscales of CSAI-2 to obtain a more detailed picture. Pre-competition anxiety is a commonly observed psychological state that affects athletes’ performance and mental health. While anxiety at a moderate level can motivate athletes and improve performance, anxiety at a high level can be a disturbance to athletes’ performance by distracting their focus, impairing motor skills, and reducing their ability to make decisions (Sánchez-Sánchez et al., 2023). In addition, anxiety can cause long-term psychological damage (Anwari et al., 2025). Given the increasing anxiety in high-level competitions, the management of pre-competition anxiety has become a key issue in sport psychology.

This study proposes a new chain mediation model of “flow experience–mental toughness” and makes the first attempt to examine the psychological basis of pre-competition anxiety in Tai Chi training. Theoretically, this study bridges the gap between physical training and psychological training by integrating knowledge from positive psychology and sport psychology. Practically, the results of this study provide experimental evidence to support the localized intervention strategy design based on the psychological characteristics of Chinese athletes. The significance of this study lies in the fact that it supports the national policy of “sports and medicine integration” and the strategy of promoting and developing traditional Chinese sports culture in modern society. Theorizing the psychological mechanisms of Tai Chi, one is highly likely to pay attention to the active components of Tai Chi. Our hypothesis is that the effects of the intervention can not be explained by one single factor but by the combination of several factors. (1) The experiment of slow, coordinated movements accompanied by conscious attention to the body sensations is supposed to lead to the state of flow because it requires balancing between a challenge and expertise, and a merger of action and mindfulness. (2) Controlled abdominal breathing is theorized to act directly on the autonomic nervous system and produce benefits on somatic anxiety to reduce but not eliminate cognitive anxiety, and also as an attentional anchor to reduce somatic anxiety. Background: (3) Mental toughness via skills mastery over time under a philosophical framework that emphasizes adaptability and persistence (e.g., softness to overcome hardness) is anticipated to develop mental toughness, consisting of improvement of self-efficacy and formation of a challenge-oriented mindset. In this way, Tai Chi is understood as an interdisciplinary intervention that simultaneously aims at physiological, attentional, and cognitive–affective pathways, which are relevant to athletic performance.

### 1.1. The Relationship Between Tai Chi Training and Pre-Competition Anxiety in High-Level Competitive Athletes

In modern society, pre-competition anxiety is a commonly observed psychological issue among high-level athletes working in competitive sports. It usually appears in the form of cognitive symptoms (such as fear of failure, negative self-evaluation) and somatic symptoms (such as increased heart rate, muscle tension). All of these seriously affect athletes’ performance (Anwari et al., 2025; Hudgins, 2025; Ma, 2024). Although traditional psychological interventions such as cognitive-behavioral therapy (CBT) have achieved certain effects, these interventions are difficult to popularize in real life because of the low compliance of athletes and relatively high cost (Romijn et al., 2021).

In recent years, Tai Chi, a traditional Chinese mind–body exercise with low intensity and meditative movement, has been found to have potential as an alternative for psychological training in athletes. Tai Chi is a kind of practice with a distinctive psychophysiological regulatory method (such as slow and continuous movement, deep breathing, and mindful awareness) that can relieve pre-competition anxiety in a multidimensional way (Naderi & Ebrahimi, 2025).

The practice of Tai Chi was associated with physiological calmness, which may be explained by its observed effects on the autonomic nervous system. Tai Chi induces physiological calmness by affecting the activity of the autonomic nervous system. Tai Chi’s slow, rhythmic motions have been shown to increase parasympathetic activity, reduce cortisol levels, and dampen the body’s stress response (Zou et al., 2018). In a randomized controlled trial with professional athletes, eight weeks of Tai Chi practice have been associated with a significant reduction in salivary cortisol as well as improved heart rate variability, markers of better autonomic regulation and stress resilience (Zou et al., 2018). Furthermore, the use of breathing techniques like abdominal respiration, which is frequently employed in Tai Chi, has been shown to improve vagal tone and alleviate anxiety-induced hyperventilation (Mendo et al., 2022). These physiological effects help reduce somatic anxiety and contribute to a state of calmness prior to competition. While many studies attribute these benefits to mindfulness, the present study proposes that the mechanism in elite sports is more performance-driven, involving a specific psychological chain: the “flow experience → mental toughness” pathway.

Cognitively, Tai Chi takes the principle of mindfulness in addressing anxiety. The practitioners must stick to the current moment and concentrate attention on their breath when they are implementing Tai Chi moves—a method consistent with most mindfulness-based interventions (MBIs) (D. Zhang et al., 2021). Such attention to the present minimizes common negative self-talk, ruminations, and catastrophic thoughts, the primary sources of cognitive anxiety (D. Zhang et al., 2021). Moreover, it has been demonstrated that experienced Tai Chi practitioners demonstrate greater attentional control and reduced levels of mental anxiety, which is why mindfulness is used as the basis of attentional regulation, and flow experience, and mental toughness are selected as mediators in the present study (Waelde, 2021). Although mindfulness is a basic mechanism of attentional control, it is specifically the use of flow experience and mental toughness that are chosen in the present study. This decision is based upon the hierarchy between psychological states in sport: flow experience is a situational, performance-based optimum state (state); mental toughness is a trait based on sustained resilience (trait). Explicitly, by looking at this chain, we go beyond general wellness to a particular performance-based psychological chain that is mandated in the high-stakes competition.

### 1.2. The Relationship Between Tai Chi Training, Flow Experience, and Pre-Competition Anxiety in High-Level Competitive Athletes

In addition to mindfulness explanations, the initial research shows that Tai Chi training is potentially able to regulate pre-competition anxiety in high-level competitive athletes, establishing a state of flow, a psychological phenomenon of positive emotional states and peak performance (Sun, 2023).

The first to introduce the phenomenon of flow as an ideal psychological state was Csikszentmihalyi and Csikszentmihalyi’s (1992). The various features that are related to this state of immersion are a balance between challenge/skill, clear goals, immediate feedback, hyper-focus, a perception of control, less self-awareness, alteration in time perception, and intrinsic enjoyment (Csikszentmihalyi & Csikszentmihalyi, 1992). Flow can be typically linked to high performance and mental working in a competitive sports environment (Cece et al., 2022).

There are several features in Tai Chi that allow it to be conducive to flow. Among them, the principle of guiding qi with the intention and moving the body with qi (F. Wang, 2024, 2025) is one of the most critical principles. To do this, the practitioners are required to focus on the execution of their movements at the given moment, but with a certain way of breathing. This meditative concentration enables athletes to concentrate on being present in the present moment when they are competing and minimizes the adverse effects of the future-orientated thoughts.

Moreover, the slow build-up of the moving difficulty in Tai Chi offers sportsmen a feasible training pattern. This chain of command guarantees a gradual rapport of talent and difficulty, a requirement of flow. Furthermore, the constant technological advancement means inherent motivation and the employees’ deliberation to stay in a suitable challenge zone with Tai Chi Chuan practice, and thus, the somatic controlled and self-regulated nature of athletes. Such enhanced mastery has the potential to provoke a sense of control over the body and composedness in the mastery somatic domain, two properties of the flow experience that may be partially withdrawn in the loss of mastery observed during competitive anxiety (Norsworthy et al., 2021).

In sum, based on the congruence of Tai Chi with the psychological and physical elements of flow, there are high chances that Tai Chi can be a useful intervention in reducing pre-competition anxiety. Tai Chi not only enhances performance, but also emotional resilience among athletes to competitive stress when they engage in deep focus, challenge, and self-mastery to practice Tai Chi.

### 1.3. The Relationship Between Tai Chi Training, Mental Toughness, and Pre-Competition Anxiety in High-Level Competitive Athletes

Mental toughness is a significant psychological attribute that enables the best performances of an athlete when the competitive environment is extremely stressful, and there is a demand in such situations (Çetin et al., 2025; Robazza et al., 2025). It is marked by adaptive qualities, namely emotional regulation, perseverance, self-confidence, and dealing with stress, and it has always been linked to low rates of pre-competition anxiety as well as better athlete performance outcomes (L. Wang, 2023; S. Zhang et al., 2024).

Conceptually, Tai Chi is congruent with some of the known mental toughness components in several ways. According to one of the fundamental principles of Tai Chi philosophy, known as relaxation without relenting, the idea of massage is connected with steady and unchanging movement and a particular mental characteristic of being calm and persistent in the state of stressful competition (Gandolfi et al., 2023). This stoicism comes in when the competition is high in demand.

Tai Chi also has a staged learning sequence, beginning with basic techniques and progressing to more complex routines. This gradual learning curve provides consistent moderate challenges, and prompts athletes to adopt a challenge mindset—a core tenet of mental toughness (Blakeney, 2025). Over time, this leads to improved confidence, emotional resilience, and coping. Furthermore, the overarching philosophy of “softness overcoming hardness” encourages flexibility, adaptability, and emotional calm when faced with external stressors. These characteristics are important components of coping flexibility seen in mentally tough athletes (Ahmed et al., 2022; Park et al., 2021).

Empirical studies also provided supportive evidence for the association between Tai Chi and mental toughness. For instance, Kohn et al. (2023) found that the scores of athletes’ Mental Toughness Questionnaire for 48 Items (MTQ48) significantly improved after Tai Chi training, and the two dimensions of commitment and emotional control were closely related to performance consistency.

### 1.4. Chain-Mediated Model Linking Tai Chi Training to Pre-Competition Anxiety via Flow Experience and Mental Toughness

Previous studies have explored the predictive contributions of flow experience and mental toughness separately in the Tai Chi training–pre-competition anxiety relationship; however, whether there is a sequential chain-mediated effect of both flow experience and mental toughness remains an interesting but unexplored question.

Although mindfulness has been widely examined as an important psychological outcome of Tai Chi practice, it primarily reflects a general attentional disposition (e.g., Kee, 2019; Su & Sumranpat Brady, 2025). In contrast, flow experience represents a situational and performance-specific psychological state, whereas mental toughness reflects a relatively stable resilience-related trait. By integrating flow experience and mental toughness into a sequential chain model, the present study aims to elucidate a competition-relevant psychological pathway through which Tai Chi training alleviates pre-competition anxiety.

Tai Chi is a structured form of movement, which is conceptually focused on mind and body union (Yeung et al., 2018). It is likely that practicing Tai Chi would bring about flow in athletes. Employees with frequent flow are usually believed to have higher psychological elongation and can more rapidly regain balance after other people experience stress, which is strongly linked with mental toughness (Codonhato et al., 2018). Thus, flow states in practice might not only offer motivating emotional experience to the athletes but also be fundamental foundations of resilience in the long term.

Conceivably, Tai Chi practice may assist the athletes to reduce the amount of their pre-competition anxiety by the following sequential chain-mediated process: Tai Chi practice leads to the flow experience; flow experience increases the mental toughness of the athletes; and competitive stress can be better regulated by the athletes. That proposed chain-mediated process proves that transient and enduring psychological variables interact in an interesting way, and we can understand that the idea of Tai Chi helping athletes to better regulate their anxiety in competitions is more comprehensive.

#### Hypotheses

**Figure 1:**
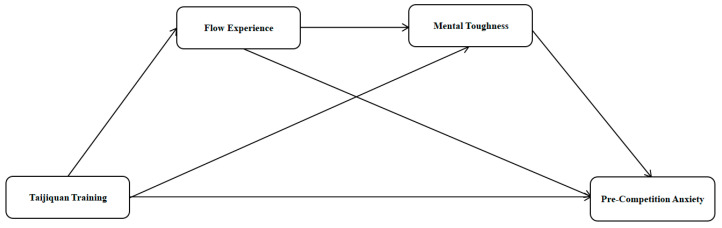
Diagram of a hypothetical model.

This study presents the four hypotheses based on the existing theories and empirical evidence to examine the effects of Tai Chi training on the psychological functioning of athletes (Figure 1).

**Hypothesis** **1** **(H1).** *Tai Chi practice is expected to reduce the performance-related anxiety among high-level competitive athletes massively prior to competitions.*

**Hypothesis** **2** **(H2).** *It is indicated that moment-to-moment mentation, in the form of flow experience, is the one to mediate the relationship (that is, Tai Chi does reduce anxiety through moment-to-moment psychological involvement).*

**Hypothesis** **3** **(H3).** *Mental toughness is another mediating variable that should explain the lessening of anxiety because Tai Chi could be used in creating a lasting psychological resilience.*

**Hypothesis** **4** **(H4).** *It is suggested that both flow experience and mental toughness mediate sequentially, and that they can be discussed as two-step mechanisms that enable Tai Chi training to reduce pre-competition anxiety.*

This model of chain mediation incorporates both the short-term experiential and the long-term dispositional variables and offers a more complete model of the psychological value of Tai Chi. In order to empirically test the hypotheses, the research will be performed using a randomized controlled design and will develop a structural equation model to analyze the direct, indirect, and chain-mediated functions of Tai Chi training on pre-competition anxiety.

## 2. Methods

### 2.1. Research Subjects

A purposive sampling method was adopted to recruit 120 high-level competitive athletes from Henan. The required sample size was a priori calculated using the G*Power software (version 3.1.9.7). Based on the meta-analysis of mind–body interventions on anxiety (for example, Zou et al., 2018), we set f^2^ = 0.15, α = 0.05, power (1 − β) = 0.85, and the result showed that the minimum required sample size should be 78. Considering the possible dropout rate of about 15%, we recruited 120 participants in total to ensure sufficient statistical power for the intended analysis. In fact, the total number of participants after three rounds of surveys was 86 due to the occurrence of injuries, diseases, other appointments, or even personal reasons.

In this study, “high-level competitive athletes” were operationally defined as athletes who (a) were registered as athletes in the national sports management system; (b) had trained for more than 8 years in a row; (c) were training for at least 20 h a week; and (d) had participated in at least three national-level competitions in the past two years, which was used as the primary inclusion criterion. They were required to participate in at least three official provincial or national competitions in the past two years, be an actively registered athlete, aged between 18 and 30 years (M = 24.1, SD = 3.5), and have no history of serious sports injuries or mental disorders. There were 45 males (52.3%) and 41 females (47.7%). In terms of sports types, 22 athletes (25.6%) were involved in track and field, 20 (23.3%) in swimming, 24 (27.9%) in wushu, and 20 (23.3%) in gymnastics. All participants participated in the study voluntarily and signed the informed consent form. The research protocol was approved by the Ethics Committee of Zhengzhou University (approval number: ZZUIRB-2024-09). Due to the possible influence of confounding variables, athletes who received psychological interventions or took psychotropic drugs in the past three months were excluded. Finally, 86 participants were randomly allocated to the experimental group and the control group, with 43 athletes in each group. T1 statistical analysis results showed that there was no significant difference between the two groups in gender (χ^2^ = 0.12, *p* = 0.73), age (t = 0.85, *p* = 0.40), or the distribution of sports types (χ^2^ = 1.03, *p* = 0.79).

### 2.2. Research Tools

#### 2.2.1. Tai Chi Training Program

A simplified version of Tai Chi, which had been modified and put together by the General Administration of Sport of China, was applied as a training protocol in this study. It is a highly standardized version that is also easy to access and convenient to train (J. X. Li et al., 2001). The program of training was drafted and carried out by a group of senior Tai Chi teachers who had more than 10 years of teaching experience, and the training program comprised three major modules, which are basic movement training (40%), complete set practice (40%), and meditation (20%). The fundamental training involved in strengthening the standardization of eight basic movements, such as Cloud Hands and Knee-Wrapping and Reversing Steps; the comprehensive set of practice concentrated on bringing forth uniformity and breathing harmony; and the meditation part element involved Positive Breathing Technique (Kabat-Zinn, 2003). The players in the experimental session underwent intensive training regimes thrice per week, which consisted of 60 min (with 10 min of relaxation and 5 min of warm-up) sessions over a duration of eight weeks. The training process was initiated with three-level quality control that involved the following: (1) video recording of every training session; (2) monthly standardized movement testing; and (3) keeping a tracking of training records, specific attendance, and subjective feedback. The control group resorted to their original training program, and they were not involved in Tai Chi practice. Control group participants were asked to continue with their habitual training programs during the study period with no deliberate attempts to increase or decrease training volume or rest period.

#### 2.2.2. Flow State Scale-2

This study employed the revised Chinese version of the Flow State Scale-2 (FSS-2), developed by Jackson and Marsh (1996), to assess athletes’ flow experience (G. Li et al., 2017). The scale comprises 36 items rated on a 5-point Likert scale (1 = not at all, 5 = fully) and evaluates nine core dimensions of the flow experience: challenge–skill balance (α = 0.82), action–consciousness integration (α = 0.79), clear goals (α = 0.81), clear feedback (α = 0.77), full attention (α = 0.83), sense of control (α = 0.80), loss of self-awareness (α = 0.78), altered sense of time (α = 0.76), and self-oriented purposefulness (α = 0.84). The total score on the scale ranges from 36 to 180, with higher scores reflecting higher levels of flow experience. In this study, the scale demonstrated good reliability, with Cronbach’s alpha coefficients of 0.87, 0.83, and 0.87, respectively. Measurements were taken at three time points: before training (T0), after 4 weeks of training (T1), and after 8 weeks of training (T2). Each measurement was administered immediately following the completion of standardized training by the athletes.

#### 2.2.3. Mental Toughness

In this study, the Chinese version of the Sport Mental Toughness Questionnaire (SMTQ), revised by Gucciardi et al. (2012), was employed to evaluate athletes’ mental toughness. The scale consists of 14 items on a 4-point Likert scale (1 = completely disagree, 4 = completely agree) and measures three core dimensions of mental toughness: confidence (α = 0.83), perseverance (α = 0.81), and resilience (α = 0.79). The total scale score ranges from 14 to 56, with higher scores indicating higher levels of mental toughness. In this study, the scale demonstrated good reliability (Cronbach’s alpha coefficients for the three dimensions were 0.88, 0.88, and 0.96, respectively). Measurements were taken at baseline, at mid-training (6 weeks), and post-training (12 weeks).

#### 2.2.4. Pre-Competition Anxiety

In this study, the Chinese revised version of the Competitive State Anxiety Inventory-2 (CSAI-2), originally developed by Martens et al. (1990), was employed to assess athletes’ levels of pre-competition anxiety. The scale consists of 27 items and is rated on a 4-point Likert scale (1 = not at all, 4 = very strongly), measuring three dimensions of pre-competition anxiety: cognitive anxiety (α = 0.86), somatic anxiety (α = 0.82), and state self-confidence (α = 0.79). It is important to note that the construct validity of the CSAI-2, particularly the stability of its three-factor structure (cognitive anxiety, somatic anxiety, and state self-confidence), has been a subject of academic discussion. Some studies have reported issues with item cross-loadings and the factorial independence of self-confidence from the anxiety dimensions. However, the CSAI-2 continues to be extensively used and validated in sport psychology research, and its framework is consistent with the Multidimensional Anxiety Theory that underpins our investigation. To ensure its appropriateness for our sample and analysis, we conducted a confirmatory factor analysis (CFA) on our baseline data. The model fit indices (e.g., CFI = 0.981, TLI = 0.977, RMSEA = 0.033) supported the acceptable fit of the three-factor model in our specific population of high-level competitive athletes. Furthermore, the high internal consistency reliability (Cronbach’s α > 0.80 for all subscales at all time points, as reported) provides additional justification for its use in this study. Therefore, despite the ongoing debate, we deemed the CSAI-2 a theoretically and psychometrically sound choice for measuring pre-competition anxiety in the present context. In the present study, the scale demonstrated good reliability (Cronbach’s alpha coefficients for the three dimensions were 0.85, 0.83, and 0.93, respectively). The CSAI-2 was administered within 24 h prior to scheduled competition events, with instructions for athletes to rate their current state of anxiety. In line with the multidimensional nature of the CSAI-2, we conducted preliminary analyses on each subscale (cognitive anxiety, somatic anxiety, and state self-confidence). For the primary hypothesis testing concerning the overarching chain-mediation model (H4), a composite score of pre-competition anxiety was used to maintain model parsimony and focus on the global effect. However, follow-up analyses examining the effects of Tai Chi training on each subscale are reported in the Results section to honor the theoretical distinctions proposed by MAT.

#### 2.2.5. Randomization Procedure

A randomized control trial design was used. The subjects given informed consent and successfully passed baseline (T0) assessment were randomly divided into the Tai Chi experimental group and the control group. An independent research assistant used a computerized random number generator that utilized a block size of 4 to obtain balanced group sizes. The order of the allocations was hidden inside numbered, dated, and blind wrapped envelopes. When the T0 assessment was performed, the principal investigator continued to open an envelope at a time in order to see the group assignment. This process also guaranteed the maintenance of allocation concealment during recruitment and enrollment. The randomization was performed at the individual level. Though the sample consisted of athletes of various sports, it was not stratified by the type of sports, though post hoc chi-square analysis confirmed that there was no significant difference between the distribution of the type of sports at the baseline of the two groups (2 = 1.03, *p* = 0.79).

#### 2.2.6. Experimental Design

**Figure 2:**
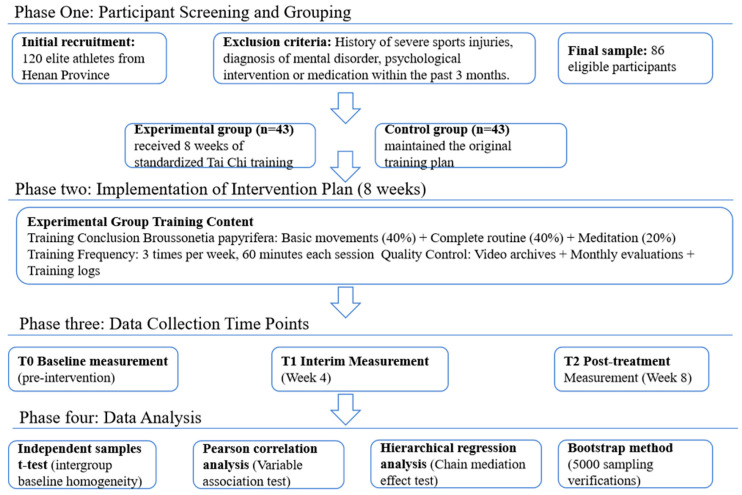
Flow chart of experimental design.

As shown in Figure 2, this study employed a randomized controlled experimental design, in which 86 high-level competitive athletes were randomly assigned to either an experimental group (n = 43) or a control group (n = 43). The experimental group underwent an 8-week standardized Tai Chi training intervention for 60 min (including 10 min of warm-up and 5 min of relaxation) three times per week, based on 24 simplified Tai Chi styles and guided by a team of professional coaches. The control group maintained its regular training program. No additional physical training, psychological intervention, or structured recovery program was introduced during the intervention period.

The Competitive State Anxiety Inventory-2 (CSAI-2) was employed as the main measurement tool, and measurements were conducted at three time points: pre-intervention (T0), 4 weeks into the intervention (T1), and 8 weeks into the intervention (T2). The Flow Status Scale-2 (FSS-2) and the Sport Mental Toughness Questionnaire (SMTQ) were additionally administered to assess mediating variables. All measures were administered using double-blind procedures by trained research assistants, and data were analyzed with SPSS 26.0 and PROCESS macros 5.0 to test chained mediation effects.

Out of the 120 individuals that were initially recruited, only 34 athletes were retained in the study, and the overall sample size was analyzed to be 86. There was no significant difference in the attrition rate between the experimental (n = 9 dropouts) and control (n = 8 dropouts) group. The rest (n = 17) of drop-outs were subsequent to the first recruitment but pre-baseline (T0) measurement and randomization, and as a result of scheduling or loss of informed consent. The most common causes of post-randomization attrition were not related to the intervention and consisted of the following: unexpected injury (n = 7), illness (n = 5), schedule conflicts with non-study competitions (n = 3), and personal reasons (n = 2). A chi-square test showed no significant difference in the percentage of dropouts between the experimental and control groups (2 = 0.06, *p* = 0.81).

## 3. Results

### 3.1. Pre-Test Homogeneity Between Experimental and Control Groups

To ensure the comparability of the experimental and control groups prior to the intervention, statistical analyses were conducted on baseline psychological characteristics. As presented in Table 1, no significant differences were found between the groups on any of the pre-test measures.

Independent sample t-tests indicated no statistically significant difference in pre-game anxiety scores (t = −0.41, *p* = 0.68, d = 0.05), flow experience scores (t = 1.08, *p* = 0.28, d = 0.13), or mental toughness scores (t = 1.82, *p* = 0.07, d = 0.21). Furthermore, all effect sizes (Cohen’s d) were below 0.30, which, according to Cohen (1988), suggests a negligible difference between groups.

These results demonstrate strong pre-intervention equivalence across the key psychological variables, providing a sound foundation for attributing post-test differences to the intervention itself.

### 3.2. Correlation Analysis

As shown in Table 2, Pearson correlation analyses revealed statistically significant associations among the variables. The group variable (experimental group = 1, control group = 0) was positively correlated at statistically significant levels with the flow experience posttest (r = 0.192, *p* < 0.01) and the mental toughness posttest (r = 0.140, *p* < 0.05), while it was negatively correlated with the pre-game anxiety posttest (r = −0.149, *p* < 0.05). The flow experience posttest was positively correlated with the mental toughness posttest (r = 0.197, *p* < 0.01), and both were negatively correlated with the pre-game anxiety posttest (flow experience: r = −0.401, *p* < 0.01; mental toughness: r = −0.228, *p* < 0.01). These correlation patterns provided preliminary support for the research hypothesis that Tai Chi training may reduce pre-competition anxiety by enhancing flow experience and mental toughness, and established a statistical basis for the subsequent chain mediation analysis.

To more rigorously assess the effect of the Tai Chi training intervention, the group variable was coded as the independent variable (1 = experimental group, 0 = control group). The post-test scores of flow experience and mental toughness were treated as mediators, while pre-competition anxiety was designated as the dependent variable. The results of the chained mediation model are presented in Table 3.

**Figure 3:**
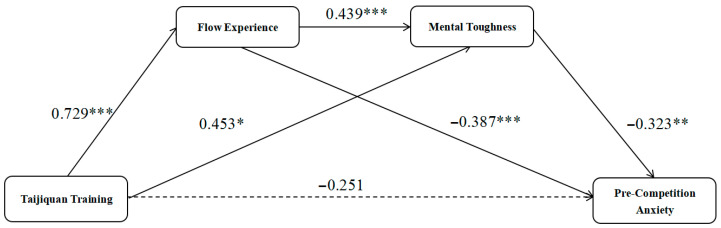
Chained mediator validation mode. Note: * *p* < 0.05; ** *p* < 0.01; *** *p* < 0.001.

As shown in Table 3 and Figure 3, hierarchical regression analysis was used to test the chain mediation effect of flow experience and psychological resilience. First, the group variable had a significant direct effect on pre-competition anxiety (β = −0.783, t = −3.927, *p* < 0.001). Second, the group variable significantly and positively predicted post-test flow experience (β = 0.729, t = 3.613, *p* < 0.001). Third, after controlling for the group variable, post-test flow experience significantly and positively predicted post-test psychological resilience (β = 0.439, t = 4.503, *p* < 0.001). Finally, when group, flow experience, and psychological resilience were entered into the model at the same time, the direct effect of group on pre-competition anxiety was no longer significant (β = −0.251, t = −1.414, *p* > 0.05), while the effects of flow experience (β = −0.387, t = −4.008, *p* < 0.001) and psychological resilience (β = −0.323, t = −3.310, *p* < 0.01) remained significant. This result meets Baron and Kenny’s (1986) criteria for testing mediation effects, indicating that Tai Chi training indirectly is associated with a reduction in pre-competition anxiety through the chain path of “enhancing flow experience → improving psychological resilience,” which supports Hypothesis H4.

As shown in Table 4, the chain mediation model was tested using the bootstrap method (with 5000 resamples). The results showed that the total effect was significant, with a value of 0.777 (95% CI [−1.173, −0.384]), while the direct effect was not significant, with a value of −0.249 (95% CI [−0.600, 0.101]), indicating that the mediation effect was fully established. Specifically, all three mediation paths reached significance: (1) the independent mediation effect through flow experience was 0.280 (95% CI [−0.524, −0.065]); (2) the independent mediation effect through psychological resilience was −0.145 (95% CI [−0.356, −0.010]); and (3) the chain mediation effect through “flow experience → psychological resilience” was −0.103 (95% CI [−0.310, −0.016]).

These results again support the research hypothesis that by improving flow experience (which in total explained 68.0% of the total mediated effect), Tai Chi training attenuates pre-competition anxiety partly through enhancing psychological resilience. There is a significant chain mediation effect (Hayes, 2018).

Although the effect size of the chain mediation path was relatively small, its 95% confidence interval did not include zero, suggesting that it was statistically significant and supported the sequential mediation process from flow experience to psychological resilience.

These results supported the research hypothesis that, by improving the flow experience (which in total explained 78.9% of the total mediated effect), Tai Chi training alleviates pre-competition anxiety partly through enhancing mental toughness. A portion of this effect was further transmitted through improving mental toughness, and there was a statistically significant serial mediation effect (Hayes, 2018). Although the effect size of the serial mediation pathway was relatively small, its 95% confidence interval did not include zero, suggesting that it was statistically significant and supported the hypothesized serial mediation process from flow experience to mental toughness.

### 3.3. The Effects of Tai Chi Training on CSAI-2 Subscales

In order to examine the distinct impact of Tai Chi training in the Multidimensional Anxiety Theory framework (Table 5), we conducted the analyses of the subscales of cognitive anxiety, somatic anxiety, and state self-confidence on the CSAI-2. All subscales also passed pre-test homogeneity (all *p* > 0.05). Repeated-measures ANOVAs (2 (Group) × 3 (Time)) showed that there were significant Group × Time interaction effects on cognitive anxiety, F(2, 168) = 5.20, *p* = 0.006, η^2^*p* = 0.058, and somatic anxiety, F(2, 168) = 4.80, *p* = 0.009, η^2^*p* = 0.054. The post hoc tests revealed that both cognitive and somatic anxiety levels improved significantly in the Tai Chi group between T0 and T2 as compared to the control group. To achieve state self-confidence, there was a significant main effect of Time, F(2, 168) = 3.60, *p* = 0.029, η^2^*p* = 0.041 and for Group, F(1, 84) = 6.50, *p* = 0.012, η^2^*p* = 0.072, with a significant main effect of Time increasing self-confidence at T0 on T2 and a significant main effect of Group increasing self-confidence at T0. These findings imply that the 8-week Tai Chi program was effective in minimizing cognitive and somatic anxiety and increasing the self-confidence of athletes.

## 4. Discussion

This study used a randomized controlled design to systematically examine the influence of Tai Chi training on pre-competition anxiety in high-level competitive athletes and to further explore the serial mediating effects of flow experience and mental toughness. By adopting a within-subject longitudinal design across three time points, the study aimed to capture changes in athletes’ psychological states under consistent pre-competition conditions. All of the hypothesized hypotheses were supported by the results, enriching the literature and offering practical references to deepen the understanding of psychological components induced by Tai Chi practice.

Firstly, the study showed that athletes’ pre-competition anxiety was significantly decreased after Tai Chi intervention (H1). This finding was consistent with previous studies that provided supporting evidence for the effectiveness of MBB interventions in reducing anxiety (Zou et al., 2018; Naderi & Ebrahimi, 2025); the effect was most apparent in the cognitive aspect of anxiety (Sánchez-Sánchez et al., 2023). As mentioned earlier, the suppression of ruminative thoughts may be due to the fact that Tai Chi practice trains athletes to maintain a positive mental framing (J. X. Li et al., 2001). Furthermore, the physiological results, heart rate variability, also suggested that athletes felt relieved in somatic anxiety symptoms after practicing Tai Chi (Mendo et al., 2022; Kohn et al., 2023). Thus, the findings of the present study indicated that the influence of Tai Chi practice on anxiety was not only limited to the mental level, but also existed in the somatic level. Consistent with Multidimensional Anxiety Theory (MAT), our supplementary analyses of the CSAI-2 subscales revealed that Tai Chi training produced beneficial effects across the different components of pre-competition anxiety. The significant reduction in cognitive anxiety aligns with the mindfulness component of Tai Chi, which trains athletes to focus on the present moment, thereby reducing negative, future-oriented thoughts and rumination (D. Zhang et al., 2021). The reduction in somatic anxiety is consistent with the documented physiological effects of Tai Chi, such as increased parasympathetic activity and improved heart rate variability, which promote physical calmness (Zou et al., 2018). Furthermore, the enhancement of state self-confidence can be attributed to the mastery experiences and improved body awareness fostered by the gradual, structured learning of Tai Chi movements, which builds a sense of competence and control. The fact that our primary chain-mediation model, which used a composite anxiety score, was significant, suggests that the pathways of “flow experience → mental toughness” are broadly applicable to the general alleviation of pre-competition distress. However, the MAT-based analysis provides a deeper, more theoretically grounded understanding of how Tai Chi operates on the specific psychological and physiological manifestations of anxiety.

Secondly, the study findings indicated that flow experience acts as a mediator of the relationship between Tai Chi practice and reduced pre-competition anxiety, which extended Csikszentmihalyi and Csikszentmihalyi’s (1992) flow theory into the context of elite sport (H2). A number of features of Tai Chi induce flow-inducing conditions (Boudreau et al., 2022): the principle of “guiding qi with intention and moving the body with qi” (J. X. Li et al., 2001); a sequence of structured physical challenges that is balanced between skill and task difficulty (Boudreau et al., 2022); fine-grained motor control that is ensured by the principle of being “loose yet unrelenting” (Cece et al., 2022); and the coordination of breath and movement, which reinforces a sense of control and integration between the action and awareness (Cece et al., 2022). While mindfulness-related attentional regulation may serve as an underlying cognitive foundation, flow experience represents a performance-oriented psychological state that is more directly linked to optimal functioning under competitive pressure.

The research also found an independent mediation effect of mental toughness (H3). This finding corroborates the previous study of Gucciardi et al. (2012) and Çetin et al. (2025) on the protective mechanism of mental toughness. Tai Chi training promotes the development of mental toughness for athletes through the following mechanisms at different levels: at the physiological level, the regulation of respiration can improve the adaptability of stress-response system (Gandolfi et al., 2023); at the psychological level, the philosophy of “softness overcomes hardness” can enhance adaptive coping strategies (Robazza et al., 2025); and at the behavioral level, the training of executive functioning through motor control can be improved (Chen et al., 2024). These mechanisms contribute to the development of mental toughness to help athletes stay emotionally stable and perform consistently in the face of competitive events. Importantly, such trait-level changes are likely to accumulate gradually through repeated exposure to structured training experiences rather than emerge immediately following short-term interventions.

The most important finding was that a serial mediation pathway passing through flow experience and mental toughness was confirmed (H4). This finding supports the view of Fredrickson (2001) and Park et al. (2021) that transient positive experiences (i.e., flow experiences) influence the development of an enduring psychological trait (i.e., mental toughness) through neuroplastic processes. That is, repeated flow experiences in Tai Chi training may influence psychological resilience by (1) enhancing the top-down regulation by prefrontal cortex over limbic system and thereby promoting emotional regulation (S. Zhang et al., 2024); (2) modulating cognitive appraisal of stress by developing challenge-oriented schemas (L. Wang, 2023); and (3) reinforcing self-efficacy by accumulating successful coping experiences (Ahmed et al., 2022). This serial mediation pathway explains the enduring effects of Tai Chi training interventions and offers a new pathway for psychological interventions in sports settings.

Interestingly, the bootstrap test showed that the effect size of the serial mediation pathway was small (0.01) but significantly different from zero. This may be due to two possible reasons: first, the effects of flow experiences on mental toughness may take time to emerge after repeated exposure (Norsworthy et al., 2021); second, the development of mental toughness is also influenced by some stable factors, such as genetic predispositions and early-life experiences (Blakeney, 2025; Codonhato et al., 2018). Nevertheless, this finding still has important theoretical significance, as it offers the first empirical evidence—in an athletic population—of a dynamic developmental pathway linking transient psychological states to enduring mental traits.

## 5. Research Limitations and Future Directions

A number of weaknesses of the current research must be mentioned. First, despite the use of a temporal criterion (within 24 h of competition) to measure pre-competition anxiety, the athletes involved in competitions of different types and levels could have affected the degree of anxiety measured on an absolute scale. Ecological accuracy can be increased in future research as competitive situations can be controlled or stratified.

Second, whereas the athletes who belonged to the control group were asked to keep following their standard training regimen without making a deliberate change in the training load or recovery period, objective measures of training intensity were not quantitatively measured. Future studies can consider objective measures of workloads in order to more thoroughly isolate the distinct effects of Tai Chi interventions.

Third, mindfulness, as a construct that was extensively studied in Tai Chi studies, was not a variable covered by the current chain mediation model. Despite the fact that the research was on the performance-based mechanisms (flow experience and mental toughness), future research can incorporate the three mechanisms, mindfulness, flow, and mental toughness, in an overall psychological framework.

To gain a better understanding of the specific mechanisms by which Tai Chi Chuan affects the mental states, future studies should be conducted on the neural mechanisms in question by utilizing neuroimaging parameters. It ought to further organize the breakdown of the main factors of Tai Chi in order to determine the active aspects of Tai Chi.

Increasing Research Scope: Improving the Analysis of Heterogeneity.

Greater sample sizes of various regions and different fields of sports should be introduced to examine the applicability of the results. At the same time, there should be an in-depth analysis of heterogeneity of intervention effects and mediating pathways between subgroups, which vary in terms of gender, age, and personality traits.

Integrated intervention programs involving Tai Chi and sports psychology interventions are recommended to be developed. They ought to carry out cross-cultural comparative studies in an attempt to ensure that the applicability of relevant theoretical frameworks to different cultural settings is established.

## 6. Theoretical and Practical Implications

In summary, the theoretical contributions of this study can be demonstrated in three aspects: (1) the extension of flow theory and application in a traditional mind–body training field (Csikszentmihalyi & Csikszentmihalyi, 1992); (2) the extension of the theoretical framework of mental toughness and clarification of the “experience–trait” transformation mechanism (Gucciardi et al., 2012); and (3) the integrative model of effects of Tai Chi training on reducing pre-competition anxiety.

In terms of practical significance, the findings of this study provide the following implications for psychological training of athletes: (1) as a feasible intervention to prevent and alleviate pre-competition anxiety, Tai Chi is recommended to be used as a systematic intervention program in the training process; (2) the intervention program should be designed to evoke flow experiences and improve training efficiency through reasonable design of movement difficulty and immediate feedback; (3) the long-term development of mental toughness can be promoted by incorporating Tai Chi’s positive cognition and philosophical ideas into training programs; and (4) the design of Tai Chi training should consider the characteristics of athletes from different sports to enhance its applicability.

In summary, based on experimental design and statistical analysis, the psychological basis of the effects of Tai Chi training on pre-competition anxiety in high-level competitive athletes was explored. The present study provided new insights and an empirical basis for further theoretical development and application in sport psychology. In the future, we should further promote the scientific implementation of mind–body training in competitive sports based on the findings of this study.

## 7. Conclusions

Through rigorous experimental design and statistical analysis, this study revealed the psychological mechanisms by which Tai Chi training influences pre-competition anxiety in high-level athletes, providing new evidence and insights for sports psychology theory and practice. Future research should build upon these findings to further deepen and expand our understanding, thereby advancing the scientific application of mind–body training in competitive sports.

## Figures and Tables

**Table 1:** Pre-test homogeneity of psychological variables between experimental and control groups (N = 86).

Variable	Group (Mean ± Standard Deviation)		*t*	*p*	*Cohen’s d*
	Experimental Group (n = 43)	Control Group (n = 43)			
Pre-match Anxiety (pre-test)	2.51 ± 0.55	2.54 ± 0.61	−0.41	0.68	0.05
Flow Experience (pre-test)	3.61 ± 1.12	3.47 ± 1.20	1.08	0.28	0.13
Mental Toughness (pre-test)	4.25 ± 0.82	4.05 ± 1.02	1.82	0.07	0.21

**Table 2:** Pearson correlation matrix of study variables, chained mediation model test.

Variable	Group	Flow Experience (Posttest)	Mental Toughness (Posttest)	Pre-Game Anxiety (Posttest)
Group	1			
Flow Experience (post-test)	0.192 **	1		
Mental Toughness (post-test)	0.140 *	0.197 **	1	
Pre-game Anxiety (post-test)	−0.149 *	−0.401 **	−0.228 **	1

Note: * *p* < 0.05; ** *p* < 0.01.

**Table 3:** Chain mediation analysis of flow experience and mental toughness.

Dependent Variable	Independent Variable	R	R2	F	β	t
Pre-competition Anxiety (post-test)	Group (1 = experimental, 0 = control)	0.394	0.155	15.423 ***	−0.783	−3.927 ***
Flow Experience (post-test)	Group (1 = experimental, 0 = control)	0.367	0.135	13.055 ***	0.729	3.613 ***
Mental Toughness (post-test)	Group (1 = experimental, 0 = control)	0.564	0.318	19.337 ***	0.453	2.339 *
	Flow Experience (Posttest)				0.439	4.503 ***
Pre-competition Anxiety (post-test)	Group (1 = experimental, 0 = control)	0.684	0.468	24.050 ***	−0.251	−1.414
	Flow Experience (Posttest)				−0.387	−4.008 ***
	Mental Toughness (Posttest)				−0.323	−3.310 **

Note: * *p* < 0.05; ** *p* < 0.01; *** *p* < 0.001.

**Table 4:** Presents the bootstrap test results for the chain mediation effects involving flow experience and mental toughness.

	Effect	95% CI		SE	Conclusion
		Lower Limit	Limit		
Total Effect	0.777	−1.173	−0.384	0.198	Significant
Direct Effect	−0.249	−0.600	0.101	0.176	Insignificant
Group → Flow Experience Posttest → Pregame Anxiety Posttest	0.280	−0.524	−0.065	0.119	Significant
Group → Mental Toughness Posttest → Pregame Anxiety Posttest	−0.145	−0.356	−0.010	0.090	Significant
Group → Flow Experience Posttest → Mental Toughness Posttest → Pregame Anxiety Posttest	−0.103	−0.310	−0.016	0.076	Significant

**Table 5:** Summary of repeated-measures ANOVA results for CSAI-2 cognitive anxiety, somatic anxiety, and state self-confidence subscales.

Subscale	Effect Type	F-Value	df	*p*-Value	Partial η^2^
Cognitive Anxiety	Group × Time Interaction	5.20	2, 168	0.006	0.058
Somatic Anxiety	Group × Time Interaction	4.80	2, 168	0.009	0.054
State Self-Confidence	Main Effect: Time	3.60	2, 168	0.029	0.041
State Self-Confidence	Main Effect: Group	6.50	1, 84	0.012	0.072

## Data Availability

The data presented in this study are available from the corresponding author upon reasonable request.
